# Simulated microgravity reduces quality of ovarian follicles and oocytes by disrupting communications of follicle cells

**DOI:** 10.1038/s41526-023-00248-5

**Published:** 2023-01-23

**Authors:** Kaixin Cheng, Xie’an Feng, Chen Yang, Chiyuan Ma, Shudong Niu, Longzhong Jia, Xuebing Yang, Jing Liang, Yingnan Bo, Kaiying Geng, Qin Li, Hua Zhang, Xiaohua Lei, Yan Zhang

**Affiliations:** 1 State Key Laboratory of Agrobiotechnology, College of Biological Sciences, China Agricultural University, Beijing, 100193 China; 2 Center for Energy Metabolism and Reproduction, Shenzhen Institutes of Advanced Technology, Chinese Academy of Sciences, Shenzhen, 518055 China

**Keywords:** Developmental biology, Cell biology, Physiology

## Abstract

Ovarian follicles are the fundamental structures that support oocyte development, and communications between oocytes and follicle somatic cells are crucial for oogenesis. However, it is unknown that whether exposure to microgravity influences cellular communications and ovarian follicle development, which might be harmful for female fertility. By 3D culturing of ovarian follicles under simulated microgravity (SMG) conditions in a rotating cell culture system, we found that SMG treatment did not affect the survival or general growth of follicles but decreased the quality of cultured follicles released oocytes. Ultrastructure detections by high-resolution imaging showed that the development of cellular communicating structures, including granulosa cell transzonal projections and oocyte microvilli, were markedly disrupted. These abnormalities caused chaotic polarity of granulosa cells (GCs) and a decrease in oocyte-secreted factors, such as Growth Differentiation Factor 9 (GDF9), which led to decreased quality of oocytes in these follicles. Therefore, the quality of oocytes was dramatically improved by the supplementations of GDF9 and NADPH-oxidase inhibitor apocynin. Together, our results suggest that exposure to simulated microgravity impairs the ultrastructure of ovarian follicles. Such impairment may affect female fertility in space environment.

## Introduction

Gravity is one of the most fundamental physical signals on Earth that regulates creatures development from cell shape to organogenesis. However, gravity is lacking in space, which leads to a series of abnormal development in various tissues^1–3^. The ovary, which is the organ that supports oocyte development, is crucial to maintain fertility and female endocrine interactions^4,5^. In the ovary, the follicles, which are composed of an oocyte and surrounding granulosa cells, are the functional units that support oogenesis^6–8^. Although it was reported that exposure to microgravity might influence female reproductive capability in space experiments^9–13^, the effects of microgravity on follicle development remain elusive.

To simulate microgravity, a rotating bioreactor, rotating cell culture system (RCCS) that can maintain cells in a controlled rotation environment to mimic the impacts of microgravity, was devised^14^. By utilizing the RCCS system, recent studies have examined the maturation of oocytes^15^ and the development of ovarian cortical pieces and ovarian follicles in a culture under the SMG condition^16^. In these studies, SMG treatment led to an abnormal meiotic spindle organization and induced cytoplasmic blebbing in cultured germinal vesicle (GV) oocytes, resulting in a failure of oocyte maturation^15^. At the tissue level, the SMG condition resulted in a decline in follicle survival in the ovarian cortical pieces and an abnormal morphology of oocytes in cultured follicles, indicating SMG also disrupted folliculogenesis^16^. However, it is still unknown how SMG disrupts folliculogenesis at the sub-cellular and molecular levels.

The ovarian follicle is composed of an oocyte and surrounding GCs. Crosstalk between oocytes and GCs, which relies on the communicating structures, is essential for proper folliculogenesis^17,18^. Previous studies have shown that transzonal projections (GC-TZPs) are derived from the inner layer GCs that connected with the oocytes to permit essential germline-somatic communication^19,20^. Our recent findings revealed that an oocyte-derived specific microvilli (Oo-Mvi) system plays a dominant role in integrating communications between oocytes and somatic cells by governing the release of oocyte-secreted factors (OSFs)^17^. These communicating structures are the convex structures of the membrane and are constructed by the F-actin-based cytoskeletal core on the cellular surface^21^, which should be sensitive to microgravity^22^. However, few studies have been conducted to investigate the interaction of GC-TZPs or Oo-Mvi related to microgravity and folliculogenesis.

In the current study, we established a 3D follicle culture system under SMG in RCCS, and analyzed the development of cultured follicles and the quality of its released oocytes under a high-resolution imaging system. Our findings indicated that SMG markedly disrupted the cytoskeleton-related communicating structures on either oocytes or GCs. In addition, the cultured follicles presented an abnormal polarity of GCs and a reduced secretion of oocyte-secreted factors such as GDF9 under SMG. These abnormal developments led to a significantly reduced oocyte quality from cultured follicles. Supplementing the SMG cultures with GDF9 or the NADPH oxidase inhibitor resulted in the reversal of these effects. In summary, our results provide systematic evidences about ultrastructural changes in follicle cells under simulated microgravity. These studies shed light on potential mechanisms that can prevent an impairment in the female reproductive system during spaceflight.

## Results

### 3D culture of ovarian follicles under SMG conditions

**Fig. 1:**
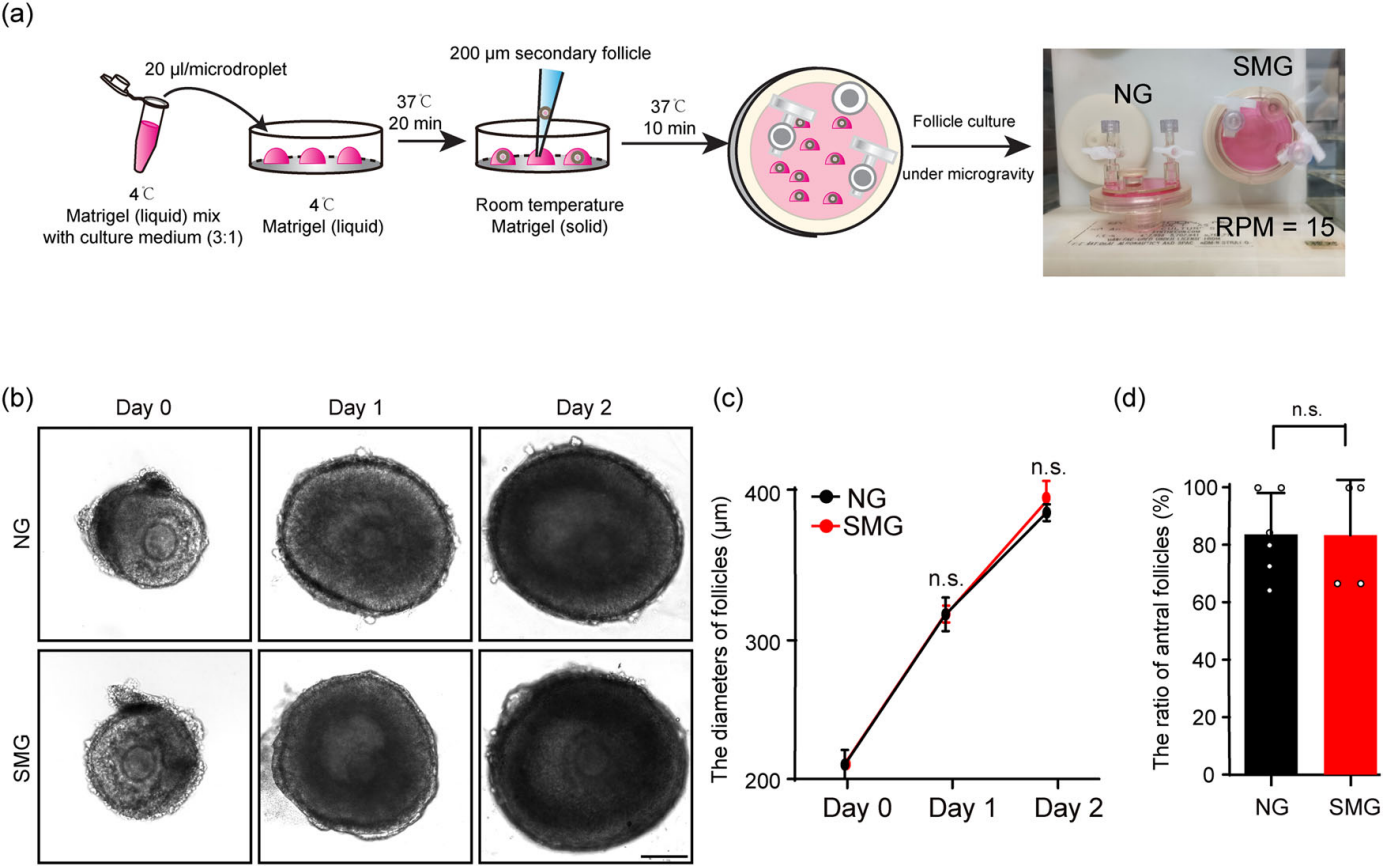
Tracing the growth of ovarian follicles in a 3D culture system under simulated microgravity condition. **a** The flowchart of the 3D ovarian follicle culture in RCCS. The follicles were seeded into a Matrigel droplet to support their growth. **b**, **c** Tracing the growth of ovarian follicles in the NG and the SMG groups in vitro (**b**), showing comparable developmental dynamics of follicles in the SMG groups (*n* = 48) compared to that in the NG group (*n* = 60) (**c**). Day 0: *p* value = 0.13. Day 1: *p* value = 0.43. Day 2: *p* value = 0.076. Scale bars: 100 μm. **d** The ratio of antral formation in the cultured follicles under different conditions, showing no significant changes of antral forming proportions of cultured follicles in the SMG group (*n* = 65) compared to that in the NG group (*n* = 73). *p* value = 0.98. Representative images are shown. Data are presented as the mean ± SD. Data were analyzed by two-tailed unpaired Student’s *t*-test and n.s. *P* ≥ 0.05.

To investigate the influences of microgravity on the development of ovarian follicles and oocytes in mice, we modified a three-dimensional follicle culture system in a RCCS to support mouse ovarian follicle development under the SMG condition^16,23^. This was achieved by seeding single follicle (about 200 μm diameter) into liquid Matrigel droplets, which were then transformed to a solid gel in the culture to support follicle growth. The droplets with follicles were cultured under the SMG condition (15 rotations per minute, RPM), or normal gravity (NG) with no rotation as the control (Fig. 1a).

With the 3D culture system, the follicles were cultured for 2 days (Fig. 1b), and the survival ratio of follicles was identical between the SMG group and the NG group (75.0% ± 5.0% in SMG v.s. 80.9% ± 1.4% in NG) (Supplementary Fig. 1), showing that SMG treatment had no effect on the survival of follicles in the culture. The diameters of the cultured follicles increased from 208.1 ± 4.45 μm to 387.3 ± 10.5 μm during 2 days of the culture in the SMG group, which was consistent with the increase in diameters of the follicles in the NG group (from 210.0 ± 8.23 μm to 384.3 ± 5.0 μm) (Fig. 1c). In addition, the ratio of antral follicles in the SMG group was similar to that in the NG group (83.3% ± 19.2% in SMG v.s. 83.6% ± 14.4% in NG) (Fig. 1d), showing the robust developmental capability of the follicles under SMG condition. These data showed that SMG treatment has no marked effect on the growth of ovarian follicles in a general observation.

### Decreased quality of oocytes after follicles developed under the SMG conditions

**Fig. 2:**
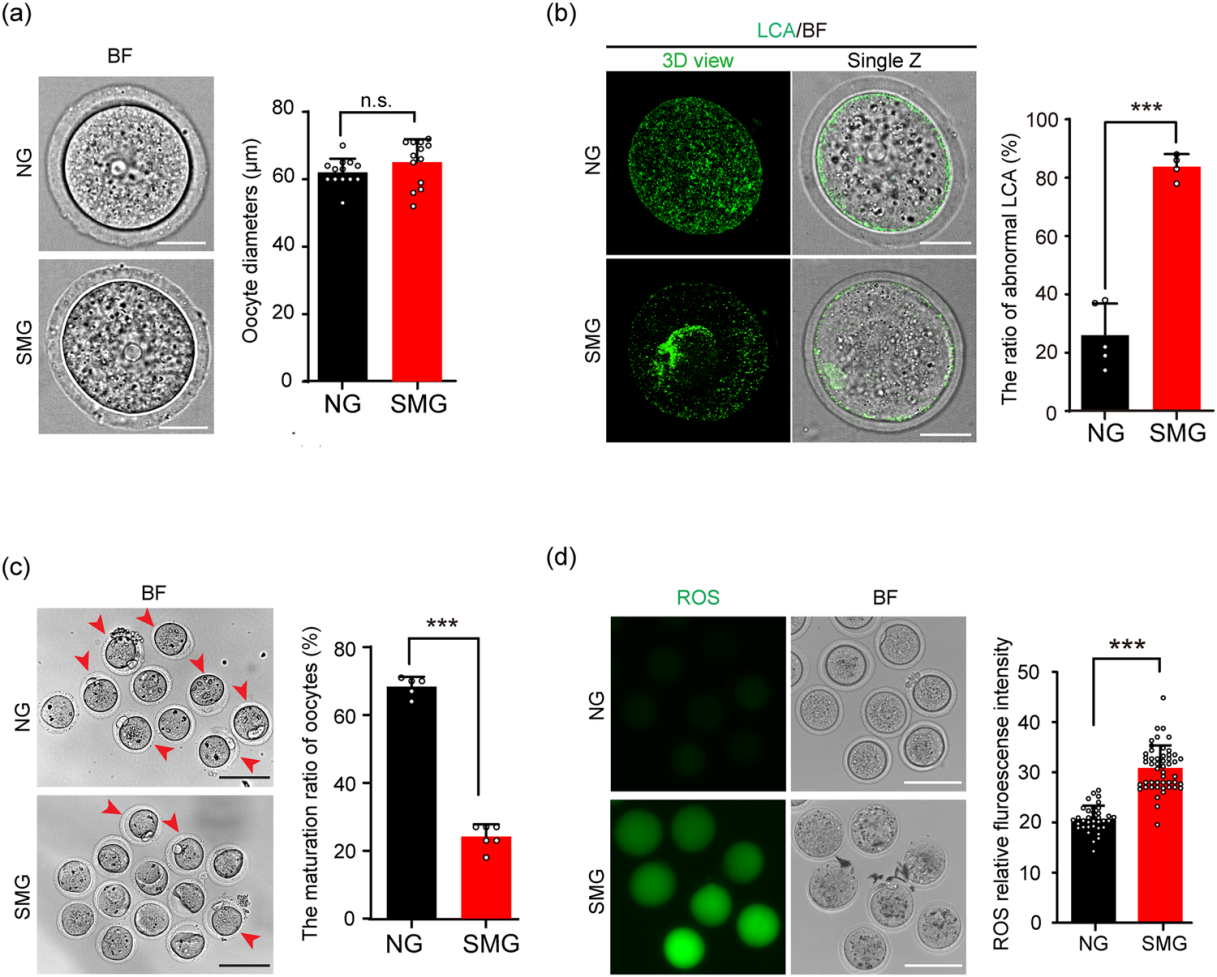
SMG treatment decreased the quality of cultured follicle released oocytes. **a** After follicles developed under SMG or NG conditions, the oocytes were isolated from the cultured follicles. No significant changes in the diameters of oocytes were seen when comparing the SMG group (*n* = 13) with the NG group (*n* = 13) after 2 days of follicle culture. *p* value = 0.18. Scale bars, 30 μm. **b** LCA (Lens Culinaris Agglutinin)-FITC immunostaining showing abnormal cortical granule distribution in the SMG group oocytes (*n* = 53) compared to that in the NG group (*n* = 59). *p* value = 0.000067. Scale bars, 30 μm. **c** Oocytes obtained from antral follicles after 16 hours of culture with LH in vitro, showing a significantly decreased ratio of PB1 (red arrowheads) in the SMG group (*n* = 74) compared to that in the NG group (*n* = 63). *p* value = 0.0000000031. Scale bars, 100 μm. **d** An increased fluorescence intensity which represented higher ROS level in oocytes of the SMG group (*n* = 50) compared to that in the NG group (*n* = 36). *p* value = 0.00000000000000000000078. Scale bars, 100 μm. Representative images are shown. Data are presented as the mean ± SD. Data were analyzed by two-tailed unpaired Student’s *t*-test and n.s. *P* ≥ 0.05, **P < 0.01, ***P < 0.001.

Because follicle development was relatively healthy under SMG condition, we next evaluated the quality of oocytes, which were released from cultured follicles in different groups. The diameter of the oocytes in the SMG group (65.1 ± 6.8 μm) was not significantly different from that in the NG group (62.1 ± 4.0 μm) (Fig. 2a), indicating that SMG treatment had no effect on oocyte size. However, a markedly abnormal distribution of the cortical granules (CG) with significantly reduced CG density and aberrated granule aggregation was observed in oocytes of the SMG group (Fig. 2b) compared to that in the NG group. Moreover, by treating the cultured follicles with luteinizing hormone (LH)^24^, we detected the ratio of first polar body (PB1) release in the oocytes from the SMG and the NG groups, and found a significant decrease in the PB1 ratio in oocytes of the SMG group compared to that of the NG group (24.2% ± 3.6% in SMG v.s. 68.4% ± 2.9% in NG) (Fig. 2c, red arrowheads), showing that the maturation of oocytes was defected under SMG conditions. According to previous studies, microgravity increased ROS production, which represented an enhancement of oxidative stress in various cell types^25^. We therefore analyzed the levels of intracellular ROS in live oocytes obtained from the follicles. Consistent with the low ratio of maturation in the SMG-treated oocytes, a remarkable increase of 2′,7′-dichlorofluorescein (DCF) fluorescence intensity, which represents a higher ROS level^26^ was also observed in oocytes from the SMG group (Fig. 2d), suggesting that SMG condition reduced the quality of oocytes.

These results demonstrated reduced quality and maturation of oocytes from SMG-treated follicles, suggesting that stimulated microgravity might affect the development of follicles at the cellular or molecular levels.

### SMG treatment disrupted the establishment of GC polarity and formation of GC projections in follicles

**Fig. 3:**
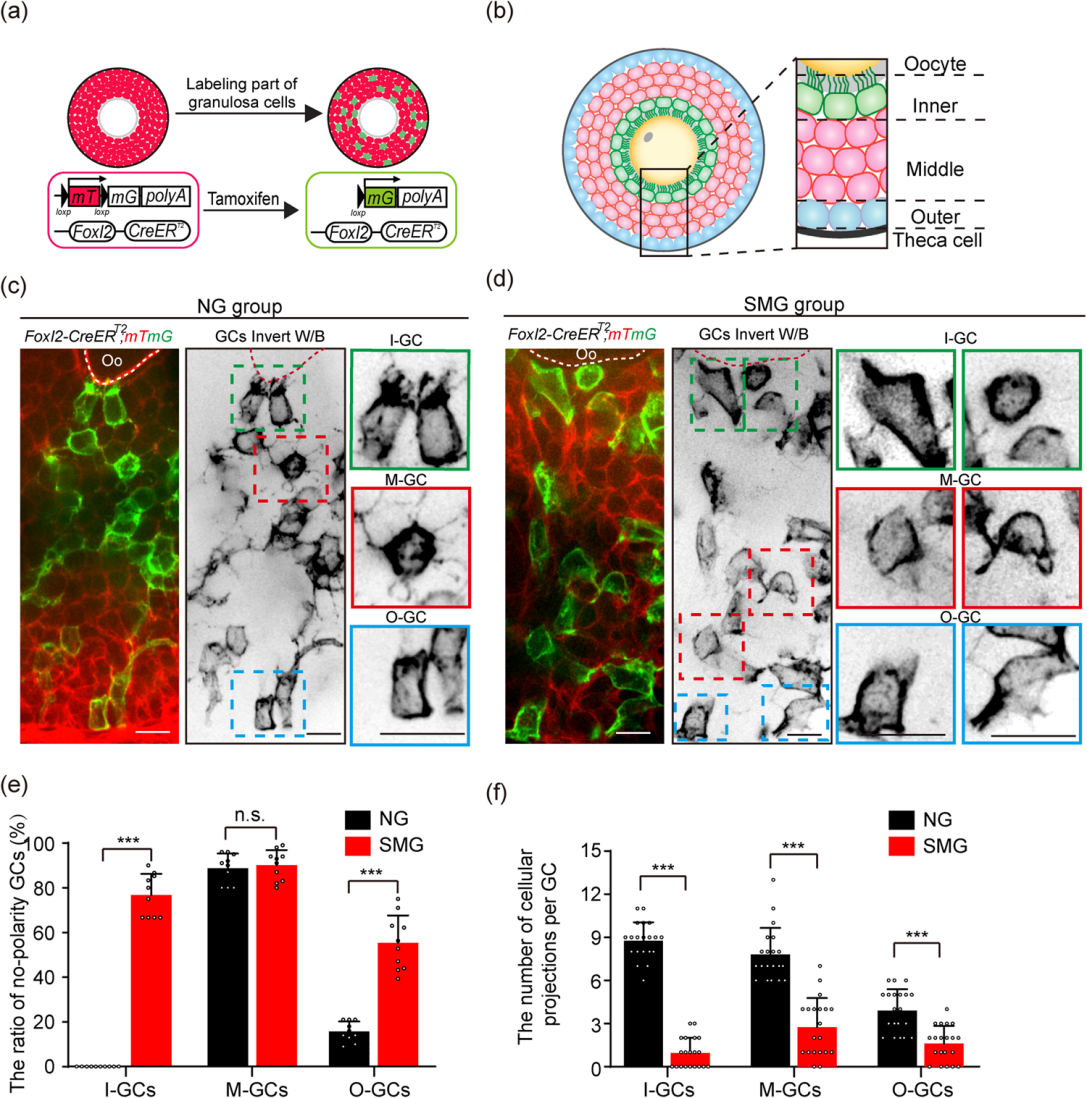
SMG treatment disrupted GC polarity and the formation of communicating structures in GCs. **a** Illustration of the strategy to induce labeling of the GCs in *Foxl2-CreER*^*T2*^*;mTmG* follicles. With a low dosage of tamoxifen treatment, membrane-localized red fluorescent protein (mT) switches to green-fluorescent protein (mG) in GCs of *Foxl2-CreER*^*T2*^*;mTmG* follicles, which allows for imaging of the cell outline of GCs under high-resolution imaging system. **b** The standard to separate the GCs in growing follicles. The inner layer of GCs was defined as the layer which directly connected to the oocyte by GC-TZPs (I-GCs, green); the middle layers of GCs was distributed in the middle multilayers position of follicle (M-GCs, pink); The outer layer of GCs was defined as the layer adjacent to the theca cells (O-GCs, blue). **c** Images of *Foxl2-CreER*^*T2*^*;mTmG* follicles showing the morphology of GCs in different regions in the NG group. I-GCs (green box) exhibited cuboidal shape with tree root-like GC-TZPs oriented toward the oocyte. Rounded M-GCs (red box) exhibited extended random cellular projections. Similar shaped cell with I-GCs, cuboidal O-GCs (blue box) extended a few cellular projections toward to M-GCs. Scale bars, 15 μm. **d** In the SMG group, both the polarity and the communicating structures on GCs were abnormal, showing a failure of GC-TZPs on I-GCs (green boxes), and dramatically reduced cellular projections on M-GCs (red boxes) and O-GCs (blue boxes). I-GCs and O-GCs exhibited a loss of polarity shape under SMG condition compared to that in the NG group. Scale bars, 15 μm. The cartoon model as shown in Supplementary Fig. 3. **e** Statistical analysis of GCs showing a significant increase in the proportion of non-polarity in I-GCs and O-GCs under SMG conditions (*n* = 10) compared to the NG group (*n* = 10). I-GCs: *p* value = 0.0000000010, M-GCs: *p* value = 0.64, O-GCs: *p* value = 0.00000082. **f** Loss of cellular projections in all layers of GCs under SMG conditions (*n* = 20) compared to that in NG conditions (*n* = 20). I-GCs: *p* value = 0.00000000000000000000064, M-GCs: *p* value = 0.00000000059, O-GCs: *p* value = 0.0000051. Representative images are shown. Data are presented as the mean ± SD. Data were analyzed by two-tailed unpaired Student’s *t*-test and n.s. *P* ≥ 0.05, ***P < 0.001. The colors were inverted to black/white (b/w) to highlight GCs in (**c**, **d**).

Because the general growth of follicles is normal, but the quality of oocytes decreased in the SMG group, we hypothesized that simulated microgravity might affect the cellular modeling and communications of GCs in the follicles. We therefore introduced a *Foxl2-CreER*^*T2*^*; mTmG* mouse model, which is capable of describing the outline of GCs with membrane-localized GFP (mG)^17,27^. Tissues from this mouse model were visualized with a high-resolution imaging system to detect the subcellular structures of GCs in the follicles (Fig. 3a). In the follicles of the NG group, we found that GCs were clearly separated to three populations with distinguished cellular characteristics, e.g. the inner layer GCs (I-GCs), which exhibited cuboidal shape with numerous stretched GC-TZPs polarity towards the oocyte (Fig. 3b, c, green box), the multi-middle layer GCs (M-GCs) with a random outline, many extended cellular projections into the surrounding cells (Fig. 3b, c, red box), and the outer layer GCs (O-GCs), which had a few cellular projections toward the M-GCs of the follicle (Fig. 3b, c, blue box). This high-resolution observation is consistent with the previous observation by TEM^28^, showing a strict cellular arrangement and polarity of GCs in follicles.

Next, we detected the GC distribution in the SMG group. Generally, we found a chaotic arrangement of GCs in the SMG-treated follicles compared to that in the NG group. Additionally, the GCs in all three populations lost their characteristics under the SMG condition. The majority of I-GCs (76.7% ± 9.5%) became round or irregular in appearance (Fig. 3d, e, green box) with a significantly decreased number of GC-TZPs compared to the NG group (0.95 ± 1.05 in SMG v.s. 8.75 ± 1.29 in NG, Fig. 3f). This result showed that the communications between GCs and oocytes were severely disrupted with SMG treatment. Similar to the I-GCs, more than half of O-GCs (55.4% ± 1.2%) lost their polarity and represented an irregular outline (Fig. 3d, e, blue box), and a reduced number of the cellular projections (1.60 ± 1.23 in SMG v.s. 3.90 ± 1.48 in NG, Fig. 3f) were also observed in those abnormal O-GCs under SMG condition. In addition, although the middle layers of GCs in the SMG group were kept in non-polarity, the number of cellular projections in each cell was significantly decreased compared with the NG group (Fig. 3c–f, red box). These results demonstrated that the simulated microgravity markedly affected the cellular polarity and the extension of the cellular projections of GCs in follicles (Supplementary Fig. 3), which disrupts the communications between GCs and the oocyte resulting in reduced quality of oocytes.

### Decline of Oo-Mvi in cultured follicles under SMG condition

**Fig. 4:**
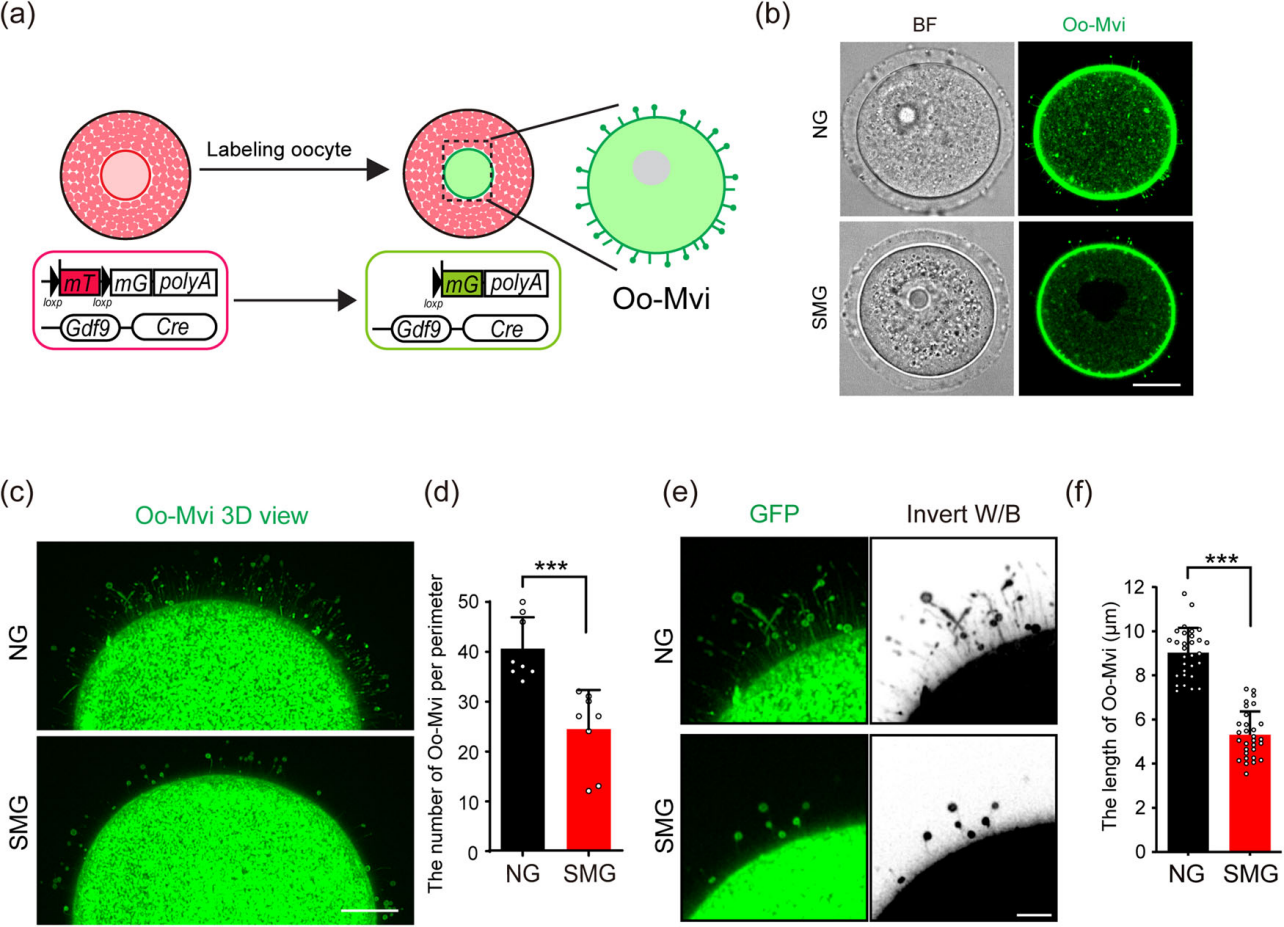
SMG treatment decreased the formation of Oo-Mvi in cultured follicles. **a** Illustration of the strategy to label the Oo-Mvi by *Gdf9-Cre;mTmG* mouse model. The membrane-localized red fluorescent protein (mT) switches to green-fluorescent protein (mG) in oocytes of *Gdf9-Cre;mTmG* mouse to label oocyte membrane morphology. **b** Images of *Gdf9-Cre;mTmG* oocytes, showing the mushroom-like Oo-Mvi with vesicle tips distributed in the zona pellucida of oocytes in both the SMG and the NG groups. Scale bars, 30 μm. **c** 3D high-resolution images showing a decreased density of Oo-Mvi on the oocytes’ surface under SMG. Scale bars, 10 μm. **d** Numbers of Oo-Mvi reduced in SMG oocytes (*n* = 8) compared to that in NG (*n* = 8), showing a significantly reduced number of Oo-Mvi on oocytes in follicles after SMG treatment. *p* value = 0.00052. **e** High magnification showing that the length of Oo-Mvi in the SMG group was shorter than that in the NG group. Scale bars, 5 μm. **f** Quantification of the length of Oo-Mvi confirmed a dramatic decrease in the SMG group (*n* = 30) compared to that in the NG group (*n* = 30). *p* value = 0.00000000000000000052. The colors were inverted to black/white (b/w) to highlight Oo-Mvi in (**e**). Representative images are shown. Data is presented as the mean ± SD. Data were analyzed by two-tailed unpaired Student’s *t*-test and ***P < 0.001.

The communications between oocytes and GCs are determined by both the oocyte-specific Oo-Mvi and the TZPs in GCs^17,18^. We next investigated whether the formation of Oo-Mvi was affected under SMG condition. Following our previous study, we crossed the *mTmG* mice with *Gdf9-Cre* mice to label the cell surface of oocytes by membrane GFP^17^ (Fig. 4a). The Oo-Mvi on oocytes from the follicles after 2 days of culturing under SMG or NG conditions were detected (Fig. 4b). As shown in Fig. 4c, our high-resolution imaging detections showed that a normal density of Oo-Mvi with typical mushroom structures was observed on oocytes from follicles in the NG group. In sharp contrast, a marked decrease in Oo-Mvi density was found on oocytes from follicles under SMG conditions (Fig. 4c). This result was confirmed by Oo-Mvi counting revealing a significantly decreased number of Oo-Mvi in the SMG group compared to that of the control oocytes in the NG group (24.5 ± 7.8 in SMG v.s. 40.6 ± 6.3 in NG, Fig. 4d). This data clearly showed that simulated microgravity also disrupts the formation of oocyte communicating structures Oo-Mvi, which could account for the low quality of oocytes seen in the SMG group.

Oo-Mvi plays a crucial regulating role for the orderly release of OSFs, and the efficiency of OSF release for each Oo-Mvi is determined by the length and the size of vesicles on the tip of Oo-Mvi^17^. We therefore measured and compared the length of Oo-Mvi and the size of Oo-Mvi vesicles after culturing in SMG versus NG. We found that although the average length of Oo-Mvi on oocytes of the SMG group was shorter than that on oocytes in the NG group (Fig. 4e, f), the average diameter of vesicles on the tip of Oo-Mvi was comparable on oocytes in the SMG and the NG groups (Supplementary Fig. 3a–b). These results suggest that the SMG only affects the formation of communicating structures, but has no marked effect on the function of these structures such as the enrichment of OSFs in Oo-Mvi.

### Supplying OSFs or Apocynin to cultured follicles improved the oocyte quality under SMG conditions

**Fig. 5:**
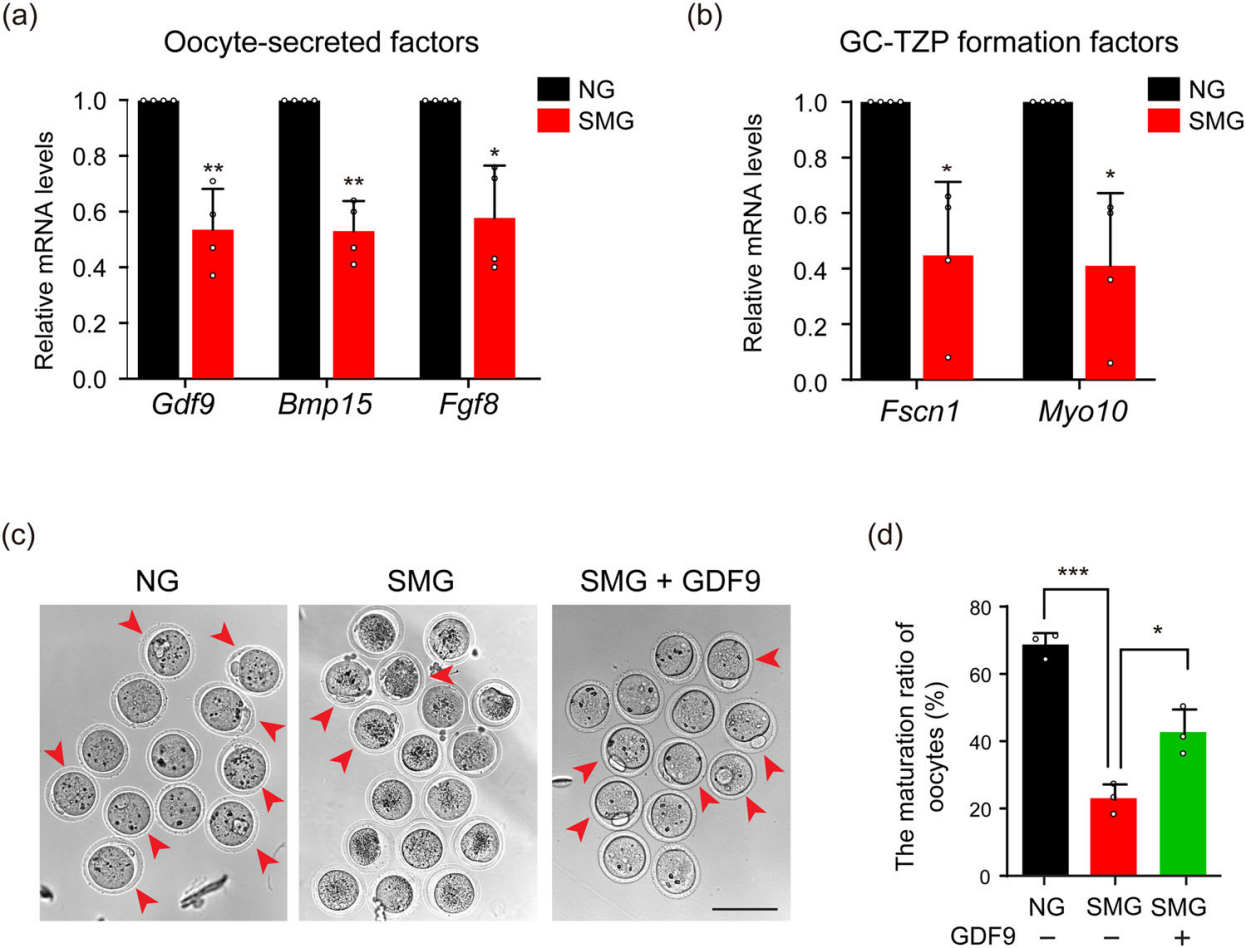
Supplying OSFs rescued the damage of oocytes by SMG treatment. **a** Relative mRNA levels of *Gdf9, Bmp15*, and *Fgf8* under the SMG or the NG group, showing a decreased expression of OSFs in the SMG group (*n* = 4). *Gdf9:* *p* value = 0.0081, *Bmp15:* *p* value = 0.0032, *Fgf8:* *p* value = 0.021. **b** Relative mRNA levels of *Fscn1* and *Myo10* under the SMG or the NG group, showing that the expression of GC-TZP forming related genes was downregulated after SMG treatment (*n* = 4). *Fscn1:* p value = 0.025, *Myo10:* p value = 0.021. **c** Supplying GDF9 increased the PB1 ratio of oocytes (red arrowheads) from SMG treated follicles. Scale bars, 100 μm. **d** The ratio of PB1 in different groups, showing that the GDF9 supplement significantly increased the maturation of oocytes (*n* = 38 in NG, *n* = 35 in SMG and *n* = 61 in SMG + GDF9). NG v.s. SMG: *p* value = 0.00021, SMG v.s. SMG + GDF9: *p* value = 0.021. Representative images of oocytes are shown. Data are presented as the mean ± SD. Data were analyzed by two-tailed unpaired Student’s *t*-test in (**a**, **b**) and two-way ANOVA in (**d**). n.s. *P* ≥ 0.05, *P < 0.05, **P < 0.01, ***P < 0.001.

Our findings demonstrate that SMG disrupts the formation of Oo-Mvi, therefore it may affect the release of OSFs in the follicles. We next detected the expression levels of several well-studied OSFs, including *Gdf9*^29,30^, *Bmp15*^30,31^ and *Fgf8*^32^ in the cultured follicles. We found that the mRNA levels of all detected OSFs were significantly reduced in follicles of the SMG group compared to that in the NG group (Fig. 5a, *Gdf9*: 0.54 ± 0.15 times, *Bmp15*: 0.53 ± 0.11 times and *Fgf8*: 0.58 ± 0.19 times), showing damage to oocytes in the cultured follicles under the SMG condition. Furthermore, we detected the cytoskeleton-related genes *Myo10*^33^ and *Fscn1*^34^, which were reported to control the formation of GC-TZPs through the regulation of OSFs in GCs^19^. As expected, we found a dramatic decrease in the expression of both *Myo10* (0.41 ± 0.26 times) and *Fscn1* (0.45 ± 0.26 times) in the follicles of the SMG group compared to that in the NG group (Fig. 5b). These results indicated that simulated microgravity caused the decline in the expression of two genes regulating connecting structures of the cytoskeleton. These abnormalities could lead to an insufficient release of OSFs to support follicluogenesis.

To confirm our hypothesis, we tested whether supplying OSFs to the cultured follicles could rescue the damage of follicles and oocytes under SMG condition. Based on our previous finding, supplying GDF9 stimulates the formation of GC-TZPs and improves follicle development^17,19,35^. We therefore cultured ovarian follicles with or without GDF9 under SMG conditions and examined the ratio of oocyte maturation in different groups. As expected, the ratio of PB1 in oocytes significantly increased from 22.8% ± 2.7% in follicles in the SMG group to 42.3% ± 4.1% after 2 days of GDF9 treatment (500 ng/mL) (Fig. 5c, d, red arrowheads). These results demonstrate that supplying OSF is efficient to rescue the defect of oocyte quality in cultured follicles under SMG.

**Fig. 6:**
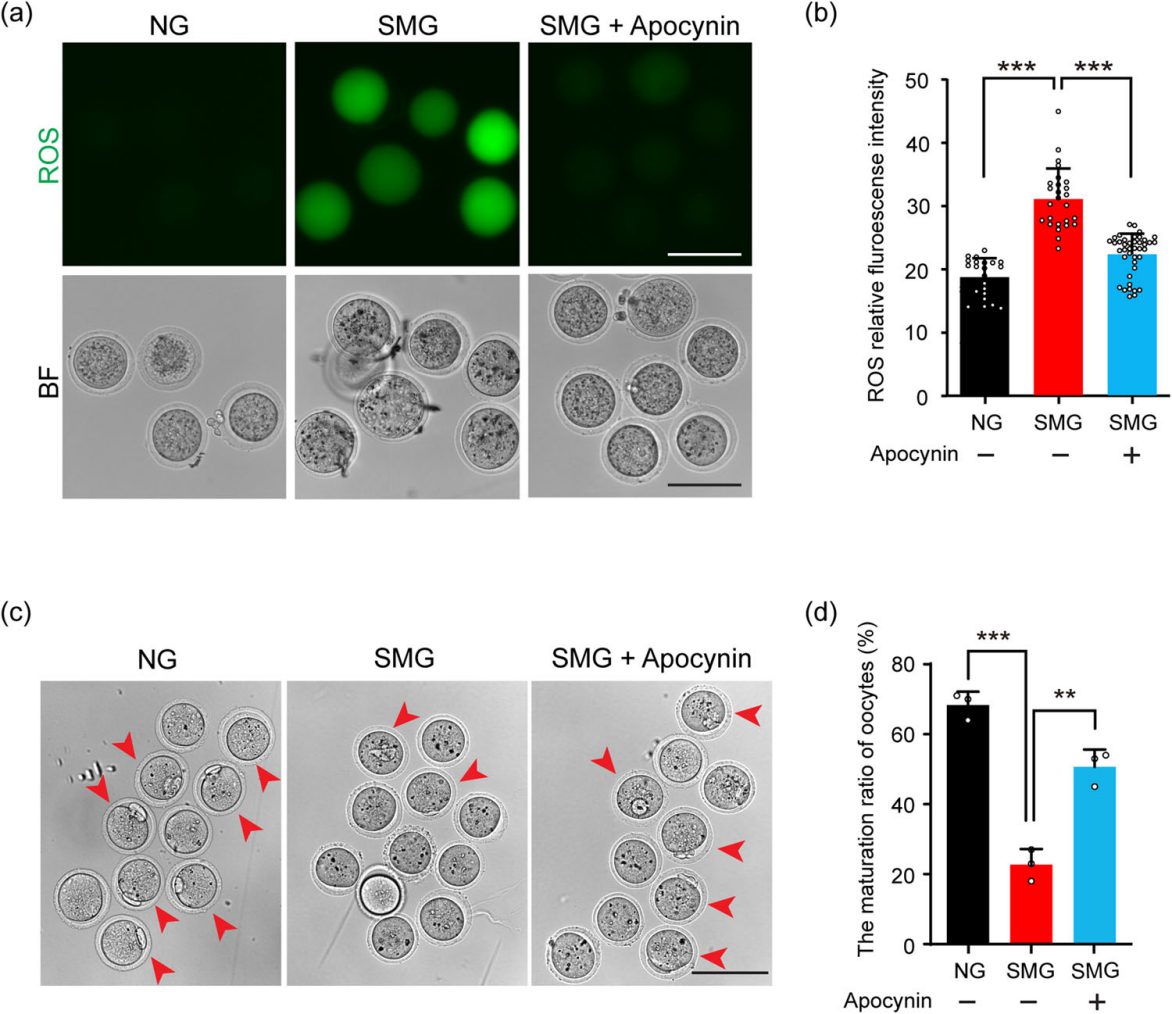
Apocynin rescued SMG-related oocyte damage by decreasing the ROS level. **a** After follicle culturing with Apocynin under SMG condition, the follicle-released oocytes showed a dramatically decreased ROS level compared to the oocytes without Apocynin. Scale bars, 100 μm. **b** The statistical analysis of DCF fluorescence intensity, showing a decreased ROS level in oocytes of the Apocynin group (*n* = 22 in NG, *n* = 26 in SMG and *n* = 41 in SMG + Apocynin). NG v.s. SMG: *p* value = 0.00000000000010, SMG v.s. SMG + Apocynin: *p* value = 0.000000028. **c** The ratio of PB1 (arrowheads) was markedly increased in the Apocynin-treated group compared to that in the SMG group. Scale bars, 100 μm. **d** Statistic analysis showing a significantly increased ratio of PB1 in the SMG + Apocynin group compared to that in the SMG group (*n* = 38 in NG, *n* = 35 in SMG and *n* = 43 in SMG + Apocynin). NG vs SMG: *p* value = 0.00021, SMG vs SMG + Apocynin: *p* value = 0.0019. Representative images of oocytes are shown. Data are presented as the mean ± SD. Data were analyzed by two-way ANOVA and n.s. *P* ≥ 0.05, **P < 0.01, ***P < 0.001.

Our experimental findings also showed an increased ROS level in oocytes from follicles under SMG condition, and the NADPH-oxidase inhibitor Apocynin was reported to be functional to improve the general cell viability under microgravity^36^. Therefore, we also cultured follicles with or without Apocynin (15 μg/mL) under SMG conditions to test the oocyte quality after culture. After 2 days of culturing, we found a significantly reduced fluorescent intensity of DCF in oocytes of the SMG group with Apocynin, showing a decreased ROS level in oocytes compared to that in the SMG group without Apocynin treatment (Fig. 6a, b). Moreover, the ratio of PB1 in the oocytes of the Apocynin group was significantly increased from 22.8% ± 2.7% in the SMG group to 50.7% ± 2.8% (Fig. 6c, d, red arrowheads), showing an efficient rescue of oocyte quality by Apocynin treatment during follicle development.

## Discussion

With the advances in aerospace technology, the biological effect of weightlessness on the reproductive health has received widespread attention^37,38^. Unlike males, who continuously produce sperm from spermatogonial stem cells^39^, the ovarian follicles of the female reproductive reserve cannot be renewed in adult life^40,41^. Therefore, any factors that cause ovarian follicle damage at any stage of follicular development lead to the irreversible reduction in female reproductive ability.

As the fundamental units of female reproduction, ovarian follicles are composed of a single oocyte and surrounding somatic cells^4^. Here, we investigated the development of follicles under SMG conditions with a focus on the cellular communication structures in follicle cells. We found the simulated microgravity disrupted the construction of GC polarity and affected the formation of cellular communicating structures in both the oocyte and GCs. With high-resolution single-cell imaging, our 3D imaging results clearly illustrated that the bidirectional communications between oocytes and GCs, including both Oo-Mvi and GC-TZPs were severely damaged by simulated microgravity, leading to the decline in oocyte quality. Previous studies reported that microgravity disrupted the polymerization and depolymerization processes of the main cytoskeleton polymers, especially the cortical actin, which causes the circularity of the cells by reducing cytoskeleton-generated tensions^42,43^. In addition, it has been reported that microfilament-based membrane structure filopodia tend to not form under microgravity^22,44^. This is consistent with our imaging observation that some I-GCs’ cuboidal shape changes to a circular shape, and there is a reduced length of the Oo-Mvi and a reduced number of GC-TZPs under SMG conditions, which was confirmed by the down-expression of F-actin regulator *Fscn1* and *Myo10* under SMG. Therefore, our results strongly suggest that the microgravity-related disorder of the cytoskeleton construction of follicle cells could be of major risk causing female reproductive disorders in space.

The main function of GCs is to nourish and support the development of oocytes^4^. With follicle development, the GCs proliferate to construct multi-layer structures^45^. It is well-known that the different layers of GCs exhibit distinguished molecular characteristics which construct a systematic regulating network, guaranteeing both the development of follicles and the maturing of oocytes^19,28^. In the current study, by utilizing a *Foxl2-CreER*^*T2*^*;mTmG* reporter mouse model to detect the membrane outline of GCs in different layers, we identified the cellular morphology of GCs with different localizations, and identified these three areas of GCs with distinguishable cellular features: cuboidal shape I-GCs whose GC-TZPs exhibited tree root-like structure polarity oriented and connected with oocytes; M-GCs which extended random cellular projections chaotically arranged in the middle position of a follicle; O-GCs adjacent to theca cells had few cellular projections toward M-GCs. These observations suggested that the GCs in various areas of the follicle had complex functional divisions to help orchestrate folliculogenesis. Moreover, we found that the cellular structures of GCs in all layers were disrupted by SMG. Such ovarian follicles are the typical structures that play their function by an integrative unit.

The effects of microgravity on mitochondrial stress, leading to increased ROS levels in several cell types, have been widely reported^25,46–48^. Our data showed the downregulation of actin-associated genes and an increase of ROS level in oocytes exposed to SMG, which resulted in the reduction of oocyte quality. Our results are consistent with previous studies that reported cytoskeletal disruption under SMG conditions induces ROS production and NADPH oxidase gene up-regulation by affecting mitochondrial membrane structure, oxygen consumption, and respiratory capacity^25,46,49,50^. Of note, our study also showed that adding OSFs (GDF9) or the NADPH-oxidase inhibitor Apocynin to the follicle of SMG cultures was able to restore oocyte quality, which provided preliminary results regarding the protective strategy to reduce the impairment of female reproduction in microgravity conditions. More importantly, this finding also suggests that the damage of follicles in microgravity can be prevented.

Because gravity is an acceleration, not a force, it depends on the mass of the whole organism to create the mechanical stimulation of cells on Earth that is then diminished during spaceflight^51^. Although various simulation devices have been developed and optimized, it remains difficult to realistically simulate the microgravity environment on the ground. This study shows the possibility of creating countermeasures to the possible harmful effects of spaceflight on the reproductive system of female astronauts. If the opportunities arise, it is preferable to carry out experiments in space and to compare the results using ground-based simulations. However, microgravity possibly works with space radiation to affect female reproduction during spaceflight.

## Methods

### Mice

C57BL/6 mice were from the Laboratory Animal Center of the Institute of Genetics (Beijing, China). *Gdf9-Cre*, *Foxl2-CreER*^*T2*^ and *mTmG* mice were generated as previously reported^27,52,53^. *Foxl2-CreER*^*T2*^ mice were a gift from Dr. Liu Kui. To partly label GCs, a single intraperitoneal injection of tamoxifen (75648, Sigma-Aldrich) at a dosage of 5 mg/kg body weight (BW) were given to *Foxl2-CreER*^*T2*^*;mTmG* females at postnatal day (PD) 21^54^. All mice were housed in mouse facilities under 16/8-h light/dark cycles at 26 °C with access to chow and water ad libitum. The methods were performed in accordance with relevant guidelines and regulations and approved by the Institutional Animal Care and Use Committee of China Agricultural University, No. AW80211202-3-1.

### In vitro culture of ovarian follicles

The follicles with the diameter of around 200 μm (average diameter: 210.0 ± 5.78 μm) were separated by tearing postnatal day (PD) 23 ovaries of C57BL/6 (for Fig. 1/ Fig. 2/ Fig. 5/ Fig. 6) or *Foxl2-CreER*^*T2*^*;mTmG* (for Fig. 3) *or Gdf9-Cre;mTmG* mice (for Fig. 4). To observe the growth of follicles, the follicles were cultured in Matrigel (BD, 354234) culture system as previously reported^23,55–57^. The culture medium consisted of 10 mL MEMα (Gibco, 32-571-036), 2.1 mg/mL NaHCO_3_, 5% FBS (Gibco, 10-099-141), 100 IU/mL penicillin-streptomycin (15140122, Invitrogen), 1% ITS (Sigma-Aldrich, 13146) and 10 ng/mL FSH (ovine Follicle Stimulating Hormone, NHPP). In the experimental group, a rotating cell culture system (RCCS-4D, SYNTHECON) was used to mimic some aspects of microgravity on the encapsulated follicles^14^. The underlying principle of the RCCS is that an increase in the rate of rotation will result in a decrease in the perimeter of the circular path^58^. The microgravity condition is simulated when the Matrigel/follicles eventually begin to rotate around their own axis. Previous studies have calculated this rate to be 15 rotations per minute^59,60^. Matrigel containing single follicles and not rotated was used as controls. In the GDF9-supplying experiment, we added GDF9 protein (500 ng/mL, 739-G9-010/CF, R&D) into follicle cultured medium for 2 days. In the Apocynin-treatment experiment, we added Apocynin (15 μg/mL, NSC 2146, Selleck) to the medium for the inhibition of ROS levels for 2 days. During culturing, the medium was half-changed every day, and the diameter of follicles was recorded every 24 h for 2 continuous days. Cultures were visualized using a Nikon Eclipse Ti digital fluorescence microscope in a bright field channel.

### Detection of Intracellular ROS in Oocytes

To analyze the levels of intracellular reactive oxygen species (ROS), a reactive oxygen species DCFH diacetate kit (E004-1-1, DCFH-DA, Nanjing Jiancheng Bioengineering Institute) was used to determine the ROS levels in living oocytes^61^. Denuded oocytes were incubated with DCFH-DA (1:1000) in PBS for 30 minutes at 37 °C in a 5% CO_2_ incubator. In the presence of ROS, H_2_DCF is rapidly oxidized to 2′,7′-dichlorofluorescein (DCF), of which fluorescent intensity represented ROS level in oocytes^26^. The oocytes were then washed three times and examined immediately for green fluorescent signals using a Nikon Eclipse Ti digital fluorescence microscope. The fluorescence intensity of the oocytes was measured using ImageJ 1.42q software (NIH).

### Oocyte maturation in vitro

For the maturation of oocytes, the follicle culture medium was changed into maturation media composed of 10 mL MEMα, 2.1 mg/mL NaHCO_3_, 5% FBS, 100 IU/mL penicillin-streptomycin, 1% ITS, and 1 μg/mL LH (ovine luteinizing hormone, NHPP) after 2 days in culture. After LH treatment for 16 h at 37 °C, 5% CO_2_, oocytes were gently removed from the follicles by a syringe needle. The polar body 1 stage of oocytes was counted as an indicator of oocyte maturation^62^.

### High-resolution imaging of follicles and isolated oocytes to detect subcellular structure

All high-resolution images of follicles and isolated oocytes were acquired using an Andor Dragonfly spinning-disc confocal microscope equipped with a ×40 or ×63 objectives, a scientific complementary metal-oxide semiconductor (sCMOS) camera (Andor Zyla 4.2), and the 488-nm (mG) and 568-nm (mT) lines of the Andor Integrated Laser Engine (ILE) system with a spinning-disc confocal scan head (Andor Dragonfly 500). Images were acquired by Fusion 2.1 software.

To identify GCs polarity, follicles were isolated from the ovaries of *Foxl2-CreER*^*T2*^*;mTmG* females following 48 h of tamoxifen treatment. After 2 days in culture, the follicles were collected from Matrigel and fixed in 4% PFA (Paraformaldehyde) in PBS for 1 h. The follicles were then transferred to clearing medium C_e_3D via mouth pipetting and incubated in the dark at room temperature on a rotor for 24 hours, as described previously^63^. The cleared follicles were embedded in a 35 mm dish with a 14 mm glass bottom (Cellvis, D35-14-517 1-N) containing fresh clearing solution and tightly covered by a coverslip. Confocal imaging was acquired with Z-step 0.6 μm for 150 μm (63× objective).

In order to quantify the polarity of granulosa cells under SMG conditions, we defined polarity GCs and non-polarity GCs according to cell morphology and localization. Polarity GCs in the inner layer of secondary follicles showed cuboidal cell shape with TZPs projecting into the zona pellucida. The outer layer of GCs adjacent to the theca cells showed cuboidal cell shape with few cellular projections towards the middle layers of GCs. Non-polarity GCs refer to those GCs that exhibit round or irregular shape. By this standard, we counted the number of non-polarity of GCs in three layers respectively to analyze the ratio of defects in polarity GCs. For every group, we counted the polarity of 50-100 cells per layers of *Foxl2-Cre*^*T2*^*;mTmG* secondary follicle (*n* = 10 follicles) as shown in Fig. 3e.

To evaluate the loss of cellular projections or TZPs of single GC of follicles exposed to SMG, we counted the number of cellular projections or TZPs of single GC from 8 μm tissue light-sections of *Foxl2-Cre*^*T2*^*;mTmG* follicles (*n* = 20 GCs of per layer from 10 secondary follicles of each groups) as shown in Fig. 3f.

To observe the Oo-Mvi in oocytes, denuded oocytes at the GV stages of antral follicles were collected from the NG or the SMG groups after 2 days culture by tearing the follicles of *GDF9-Cre;mTmG* females with a syringe needle. The oocytes were transferred via mouth pipetting ~20 μL minimum MEMα-FBS-ITS medium: MEMα with 10% FBS and 1% ITS, covered with mineral oil (Sigma-Aldrich, M8410) and photographed in a living cell workstation (Okolab) at 37 °C, 5% CO_2_. Images were typically acquired with an optical slice thickness of 0.5 μm and covered ~40 μm of oocytes.

### The Cortical Granules Staining by LCA (Lens Culinaris Agglutinin)-FITC

To detect the cortical granules, oocytes of the NG and the SMG groups at the GV stage were fixed in 4% PFA in PBS for 15 min at room temperature (25 °C), followed by treatment with 0.5% Triton X-100 for 20 min. Oocytes were subsequently incubated in PBS supplemented with 1 mg/mL BSA (Sigma-Aldrich, V900933) for 1 h. After staining with LCA (Lens Culinaris Agglutinin)-FITC for 2 h (1:100 dissolved in PBS, L32475, ThermoFisher Scientific) at room temperature, the oocytes were washed three times in PBS and imaged using an Andor Dragonfly spinning-disc confocal microscope as previously described^64^. All steps were at room temperature (25 °C).

### Gene expression analysis

To detect the gene expression of follicles after culturing, the mRNA of follicles from the NG and the SMG groups was extracted by TRIZOL Reagent (Thermo-Ambion, 15596018) according to the manufacturer’s protocol. The quantity and quality of the total RNA were determined using a Nanodrop (Thermo Scientific). Reverse transcription (TAKARA, RR047Q) was performed using 0.5 μg total RNA per sample. QRT-PCR reactions were performed in 96-well plates (Applied Biosystems, 4316813) in 10 μL reaction volumes and analyzed by an Applied Biosystems 7500 Real-Time PCR System (Applied Biosystems, 4472908) using the following parameters: 10 minutes at 95 °C, followed by 40 cycles of 15 seconds at 95 °C and 20 seconds at 50 °C and 30 seconds at 72 °C. Data were normalized to β-actin. The primer list is provided in Supplementary Table 1.

### Statistical analysis

All experiments were repeated at least three times and representative results are shown. Data are presented as the mean ± standard deviation (SD) of each experiment. Data were analyzed by Student’s *t*-test or two-way ANOVA and were considered statistically significant at *P* < 0.05. P is indicated as follows: *(*P* < 0.05), **(*P* < 0.01), ***(*P* < 0.001), and n.s. (not significant, *P* ≥ 0.05). Statistics and graphs were obtained using Prism 5 (GraphPad Software, La Jolla).

### Reporting Summary

Further information on research design is available in the Nature Research Reporting Summary linked to this article.

## Supplementary information

Supplementary information

Reporting Summary Checklist

## Data Availability

The authors declare that the data supporting the findings of this study are available within the paper.
