# Supplementary material for: Simulated microgravity reduces quality of ovarian follicles and oocytes by disrupting communications of follicle cells
Source: NPJ Microgravity. 2023 Jan 23;9:7. doi: 10.1038/s41526-023-00248-5 (PMC9870914; doi:10.1038/s41526-023-00248-5)
Supplement: Supplementary file 1 — Supplementary information [file 41526_2023_248_MOESM1_ESM.pdf]

Supplementary information

Simulated microgravity reduces quality of ovarian follicles and oocytes by disrupting communications of follicle cells

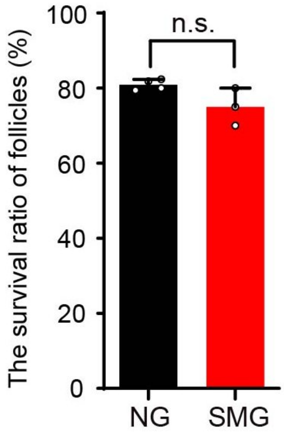

Supplementary Fig. 1 SMG treatment had no effect on the survival of follicles. The statistical analysis of the survival ratio of follicles, showing no difference in follicle survival in the SMG group (n = 36) compared to that in the NG group (n = 27). p value = 0.17. Data are presented as the mean ± SD. Data were analyzed by two-tailed unpaired Student's *t*-test and n.s. $P \geq 0.05$.

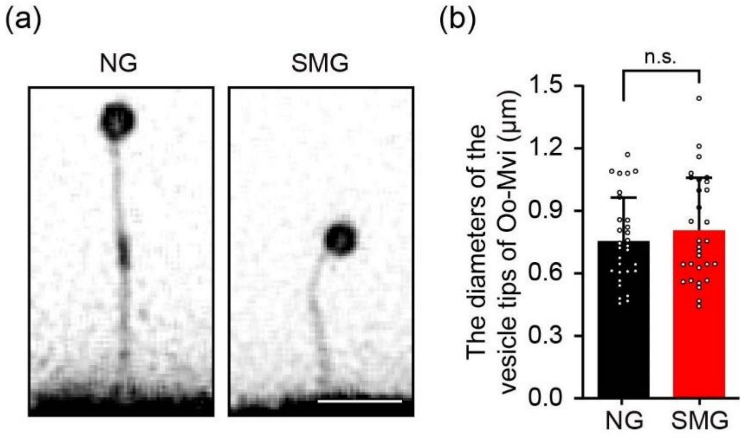

Supplementary Fig. 2 A normal development of the vesicles in Oo-Mvi under SMG conditions. a High-resolution images showing a comparable diameter of vesicles on the tip of Oo-Mvi after SMG treatment and NG treatment. Scale bars: 3 μm. **b** Quantification of vesicle diameters of Oo-Mvi showed no significant difference in vesicle size in the SMG-treated oocytes (n = 30) compared to that in the NG oocytes (n = 30). p value = 0.39. Representative images are shown. Data are presented as the mean ± SD. Data were analyzed by two-tailed unpaired Student's *t*-test and n.s. $P \geq 0.05$.

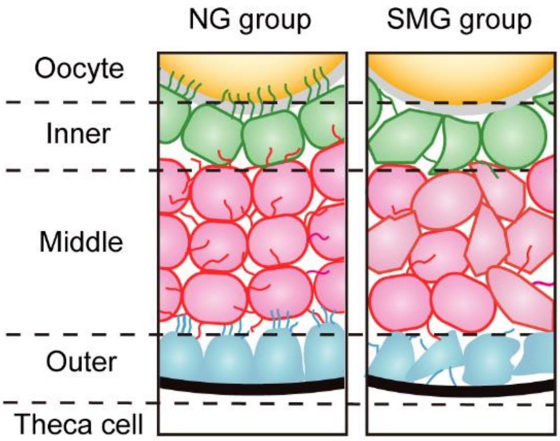

Supplementary Fig. 3 Simulated microgravity markedly affected the cellular polarity and the extension of the cellular projections of GCs in follicles. Illustration of the characteristics of GCs in three different layers of growing follicles. In the NG group, GCs in inner layer (I-GCs) are polar and protrude numerous GC-TZPs towards the oocytes (Green, left); in middle layers, GCs (M-GCs) are no polarity and extend a few random cellular projections towards surrounding cells (Red, left); GCs in outer layer (O-GCs) have a few cellular projections towards the middle layers of GCs (Blue, left). However, compared to GCs in the NG group, I-GCs (Green, right) and O-GCs (Blue, right) of the SMG group exhibit a loss of polarity shape and the shape of numerous GCs in three layers is irregular. I-GCs show a failure of GC-TZPs compared to the NG group (Red, right).

Supplementary Table 1. Primer sequences used for QRT-PCR analyses

Gene	Sequence
β-actin	GTGACGTTGACATCCGTAAAGA
	GCCGGACTCATCGTACTCC
Gdf9	TCACCTCTACAATACCGTCCGG
	GAGCAAGTGTTCCATGGCAGTC
Bmp15	GCACGATTGGAGCGAAAATG
	CGTACGCTACCTGGTTTGATGC
Fgf8	CAGGTCTCTACATCTGCATGAACAA
	TCTCCAGCACGATCTCTGTGAATA
Fscn1	AGAACGCCAGCTGCTACTTT
	CGAGGAATCACTACCCACCG
Myo10	TCCAGACAGACTATGGGCAGG
	GGAAGCCATGTCGTCCACG
