# Supplementary material for: Simulated microgravity reduces quality of ovarian follicles and oocytes by disrupting communications of follicle cells
Source: NPJ Microgravity. 2023 Jan 23;9:7. doi: 10.1038/s41526-023-00248-5 (PMC9870914; doi:10.1038/s41526-023-00248-5)
Supplement: Supplementary file 2 — Reporting Summary Checklist [file 41526_2023_248_MOESM2_ESM.pdf]

nature portfolio

Corresponding author(s): Yan Zhang

Last updated by author(s): Dec 13, 2022

Reporting Summary

Nature Portfolio wishes to improve the reproducibility of the work that we publish. This form provides structure for consistency and transparency in reporting. For further information on Nature Portfolio policies, see our Editorial Policies and the Editorial Policy Checklist.

Statistics

For all statistical analyses, confirm that the following items are present in the figure legend, table legend, main text, or Methods section.

n/a	Confirmed	
☐	☒	The exact sample size (n) for each experimental group/condition, given as a discrete number and unit of measurement
☐	☒	A statement on whether measurements were taken from distinct samples or whether the same sample was measured repeatedly
☐	☒	The statistical test(s) used AND whether they are one- or two-sided<br>*Only common tests should be described solely by name; describe more complex techniques in the Methods section.*
☐	☒	A description of all covariates tested
☐	☒	A description of any assumptions or corrections, such as tests of normality and adjustment for multiple comparisons
☐	☒	A full description of the statistical parameters including central tendency (e.g. means) or other basic estimates (e.g. regression coefficient) AND variation (e.g. standard deviation) or associated estimates of uncertainty (e.g. confidence intervals)
☐	☒	For null hypothesis testing, the test statistic (e.g. F, t, r) with confidence intervals, effect sizes, degrees of freedom and P value noted<br>*Give P values as exact values whenever suitable.*
☒	☐	For Bayesian analysis, information on the choice of priors and Markov chain Monte Carlo settings
☒	☐	For hierarchical and complex designs, identification of the appropriate level for tests and full reporting of outcomes
☒	☐	Estimates of effect sizes (e.g. Cohen's d, Pearson's r), indicating how they were calculated

Our web collection on statistics for biologists contains articles on many of the points above.

Software and code

Policy information about availability of computer code

Data collection	No computer code involved in the manuscripts.
Data analysis	No computer code involved in the manuscripts.

For manuscripts utilizing custom algorithms or software that are central to the research but not yet described in published literature, software must be made available to editors and reviewers. We strongly encourage code deposition in a community repository (e.g. GitHub). See the Nature Portfolio guidelines for submitting code & software for further information.

Data

Policy information about availability of data

All manuscripts must include a data availability statement. This statement should provide the following information, where applicable:

- Accession codes, unique identifiers, or web links for publicly available datasets
- A description of any restrictions on data availability
- For clinical datasets or third party data, please ensure that the statement adheres to our policy

The authors declare that the data supporting the findings of this study are available within the paper and its supplementary information files.

Human research participants

Policy information about studies involving human research participants and Sex and Gender in Research.

Reporting on sex and gender	NA
Population characteristics	NA
Recruitment	NA
Ethics oversight	NA

Note that full information on the approval of the study protocol must also be provided in the manuscript.

Field-specific reporting

Please select the one below that is the best fit for your research. If you are not sure, read the appropriate sections before making your selection.

☒ Life sciences ☐ Behavioural & social sciences ☐ Ecological, evolutionary & environmental sciences

For a reference copy of the document with all sections, see nature.com/documents/nr-reporting-summary-flat.pdf

Life sciences study design

All studies must disclose on these points even when the disclosure is negative.

Sample size	All experiments were repeated at least three time and showed representative results. Data are presented as the mean ± standard deviation (SD) of each experiment. Data were analyzed by Student's t-test or two-way ANOVA, and were considered statistically significant at $P < 0.05$. P is indicated as follows: *($P < 0.05$), **($P < 0.01$), ***($P < 0.001$), and n.s. (not significant, $P \geq 0.05$). Statistics and graphs were obtained using Prism 5 (GraphPad Software, La Jolla).
Data exclusions	No data were excluded.
Replication	All experiments were repeated at least three times using different mice.
Randomization	Samples, organisms, and participants were randomly allocated into experimental groups.
Blinding	investigators were blinded to group allocation during data collection or analysis.

Reporting for specific materials, systems and methods

We require information from authors about some types of materials, experimental systems and methods used in many studies. Here, indicate whether each material, system or method listed is relevant to your study. If you are not sure if a list item applies to your research, read the appropriate section before selecting a response.

Materials & experimental systems

n/a	Involved in the study	
☒	☐	Antibodies
☒	☐	Eukaryotic cell lines
☒	☐	Palaeontology and archaeology
☐	☒	Animals and other organisms
☒	☐	Clinical data
☒	☐	Dual use research of concern

Methods

n/a	Involved in the study	
☒	☐	ChIP-seq
☒	☐	Flow cytometry
☒	☐	MRI-based neuroimaging

Animals and other research organisms

Policy information about studies involving animals; ARRIVE guidelines recommended for reporting animal research, and Sex and Gender in Research

Laboratory animals	C57BL/6 mice were from the Laboratory Animal Center of the Institute of Genetics (Beijing, China). Gdf9-Cre, Foxl2-CreERT2, and mTmG mice were generated as previously reported (Lan and Z.-J., 2004; Muzumdar et al., 2010; Zheng et al., 2014). To partly label

	GCs, a single intraperitoneal injection of tamoxifen (75648, Sigma-Aldrich) at a dosage of 5 mg/kg body weight (BW) were given to Foxl2-CreERT2;mTmG females at PD21(Hayashi and Mcmahon, 2002).
Wild animals	The study did not involve wild animals.
Reporting on sex	The study is only about the development of ovarian follicles and oocytes in females. Therefore, the findings in this study only apply for female.
Field-collected samples	The study did not involve field-collected samples.
Ethics oversight	All mice were housed in mouse facilities under 16/8-h light/dark cycles at 26°C with access to chow and water at libitum. The animal experiments conformed to the guidelines and regulatory standards of the Institutional Animal Care and Use Committee of China Agricultural University, No. AW80211202-3-1.

Note that full information on the approval of the study protocol must also be provided in the manuscript.
